# The Diagnosis of Sepsis-Associated Encephalopathy Using Biomarkers: Are We There Yet?

**DOI:** 10.1055/s-0045-1808060

**Published:** 2025-05-06

**Authors:** Florin Scarlatescu, Ecaterina Scarlatescu, Dana R. Tomescu, Daniela Bartos

**Affiliations:** 1 Department of Neurology, Clinical Emergency Hospital, Bucharest, Romania; 2 University of Medicine and Pharmacy Carol Davila, Bucharest, Romania; 3 Department of Anaesthesia and Intensive Care, Fundeni Clinical Institute, Bucharest, Romania

**Keywords:** encephalopathy, sepsis, biomarkers, diagnosis

## Abstract

Sepsis-associated encephalopathy (SAE) is a diffuse brain dysfunction that occurs in patients with sepsis in the absence of direct central nervous system infection or other causes of encephalopathy. SAE is common, occurring in up to 70% of patients with sepsis, and is linked to various clinical manifestations and significantly poorer outcomes. The diagnosis of SAE usually relies on clinical examination, which is often difficult due to confounding factors in critically ill patients. Other diagnostic tools used include electroencephalography, neuroimaging, and biomarkers. We performed a systematic search and review to synthesize all available evidence on biomarkers used for SAE diagnosis in clinical practice and highlight future directions for research. The literature search in MEDLINE identified 18 eligible studies. Biomarkers reflecting inflammation, endothelial activation and damage, astrocytic and microglial activation, neuronal injury, and metabolism changes were described, demonstrating their usefulness and potential in diagnosing and evaluating SAE. However, among different studies, the reported sensitivity and specificity of the biomarkers for diagnosing SAE varied based on the populations studied and the cutoff levels considered for each biomarker. In conclusion, biomarkers may be useful for diagnosing and predicting outcomes in SAE, but their usefulness in clinical practice remains limited for the moment. More research is needed to identify biomarkers that can improve SAE diagnosis.

## Introduction

Sepsis-associated encephalopathy (SAE) is characterized by diffuse neurological impairment caused by a dysregulated systemic response to severe infections or sepsis without direct central nervous system (CNS) infection, acute cerebral lesions, or other causes of encephalopathy. 1 The clinical manifestations of SAE are heterogeneous, ranging from mild symptoms such as confusion, hypersomnolence, or delirium to severe neurological manifestations such as stupor or coma. Depending on the clinical manifestations considered for SAE diagnosis, the reported incidence of SAE is highly variable, from 43 to 76%. 2 3 4 5 6 In a recent study including more than 4,000 septic patients from the Medical Information Mart for Intensive Care IV (MIMIC-IV) database and the eICU database, SAE was defined as a Glasgow Coma Scale (GCS) score of less than 15 or abnormal neurological findings consistent with delirium and occurred in up to 68% of patients. 7

The pathophysiology of SAE involves multiple complex mechanisms. The dysregulated immune response in sepsis leads to the exaggerated release of proinflammatory cytokines and generalized endothelial damage, including the endothelial cells forming the blood–brain barrier (BBB). This results in the activation of microglia and damage to neuronal cells and astrocytes, as immune cells and cytokines can now enter the CNS due to changes in the function of the BBB. 8 Other mechanisms contributing to SAE pathogenesis include microvascular dysfunction and hypoxic–ischemic mechanisms, oxidative stress, and metabolic or neurotransmitter disturbances. 7

The diagnosis of SAE relies on clinical assessment using scales for assessing consciousness, such as the GCS, or for assessing delirium (such as the confusion assessment method for the ICU). However, there are difficulties associated with these diagnostic methods: the clinical assessment of the confusion or consciousness score can be subjective and is also influenced by other factors often found in intensive care unit (ICU) settings, such as sedation and analgesia to facilitate ventilation or different procedures. In the absence of a screening tool or investigation, the early diagnosis of SAE is often missed, especially in patients with mild neurological impairment or hypoactive delirium. Once neurological dysfunction is identified, other diagnostic modalities for assessing SAE include neuroimaging (brain computed tomography [CT] or magnetic resonance imaging [MRI]), transcranial Doppler ultrasonography, or electroencephalography (EEG). Changes identified by neuroimaging are nonspecific and typically appear later during the infectious process, while sedation can also influence EEG results.

The early diagnosis of SAE is essential in clinical practice, as the alteration of neurological status can often represent the first clinical manifestation of organ dysfunction due to sepsis. Therefore, the correct diagnosis of neurological dysfunction can aid in the timely diagnosis of sepsis. Moreover, several studies demonstrated that the occurrence of SAE is independently associated with a worse prognosis in septic patients; thus, diagnosing SAE would allow the identification of more severe patients who might benefit from timely medical intervention, including more aggressive or invasive therapeutic measures to control the infectious process. 4 5 6 7 It can be challenging to diagnose SAE in clinical practice, as it involves ruling out other conditions such as cerebrovascular diseases, CNS infection, brain injury from trauma, brain tumors, metabolic encephalopathy, and drug side effects.

Several publications reported using biomarkers for SAE diagnosis or prediction in the last decade. This represents a novel approach for detecting SAE, practical in the cases where clinical neurological assessment or brain MRI is not feasible or when the results of other investigations are inconclusive. Identifying biomarkers for SAE diagnosis would facilitate the early identification of SAE and improve the understanding of molecular mechanisms responsible for SAE, together with possible novel therapeutic strategies. The aim of this systematic search and review is to synthesize and discuss all available evidence on the use of biomarkers for SAE diagnosis and their utility in clinical practice, as well as highlight future directions for research.

## Methods

A literature search was conducted in MEDLINE using the following Medical Subject Headings (MeSH) terms from the National Library of Medicine list of search terms: “sepsis-associated encephalopathy” (or “septic encephalopathy,” “septic neurologic dysfunction,” “sepsis-induced encephalopathy”) and “biomarker” (or “laboratory test,” “plasmatic marker,” “serological marker,” “blood test,” “diagnosis”). The search was restricted to articles in English published over the past 50 years (from 1974 to 29 February 2024). Study designs eligible for inclusion are case series, case-control studies, cross-sectional studies, prospective or retrospective cohort studies, and randomized controlled trials including adult patients. Review articles, editorials, letters to the editor, case reports, publications without an abstract, and studies, including pediatric patients and animal studies, were excluded. All cited references were reviewed to identify additional studies. The specific entry terms of the search are detailed in Supplementary File 1 (available in the online version only).

Two authors (E.S., F.S.) independently conducted the search and study selection. They also searched the reference lists of selected studies. Any disagreement was resolved by involving the other two authors.

## Summary of Evidence

### Study Selection

**Fig. 1:**
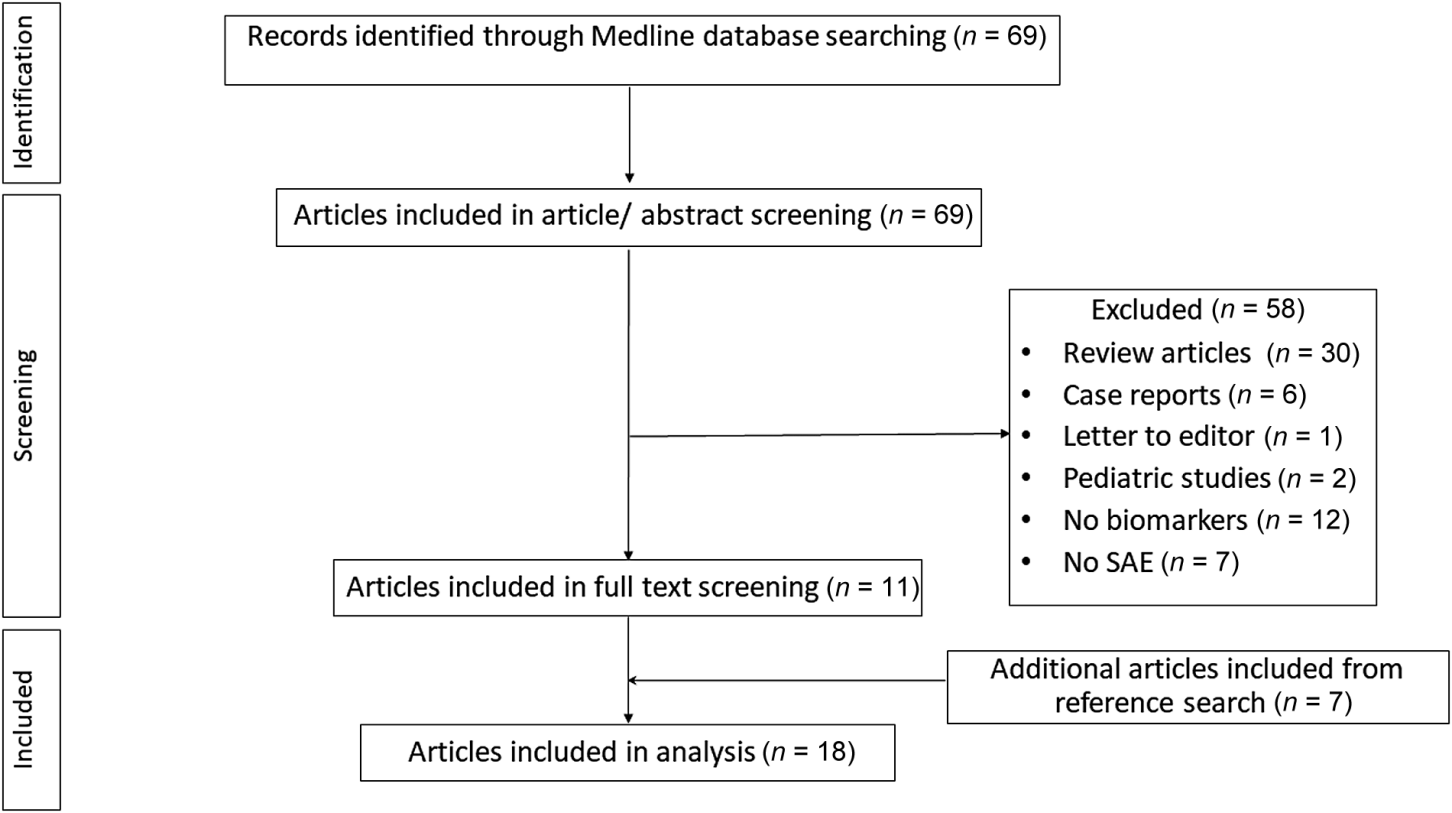
Flowchart. *n* , number

The search strategy yielded 69 references, from which 58 were excluded after a review of titles and abstracts. Seven more articles were identified from the citation search, leading to a total of 18 articles used for data extraction ( Fig. 1 ).

The oldest article selected was published in 2005, and 6 of the 18 articles were published in the last 5 years (in 2020 or after). Table 1 summarizes the different biomarkers used for SAE diagnosis. Table 2 presents the findings from the 18 articles included in this review.

**Table 1:** Biomarkers evaluated for SAE diagnosis

Biomarker	Expressed/produced by	Structure	Biomarker source	Findings
**Cytokines**				
IL-6	Macrophages	Protein	Blood, CSF	Higher levels in blood samples from critically ill septic patients with SAE than in non-SAE patients 10 Higher levels in CSF from critically ill septic patients with SAE than in non-SAE patients 11 Higher blood levels in SAE patients compared to healthy controls 13
IL-8	Macrophages, microglia, and astrocytes in brain	Protein	CSF, blood	Higher CSF levels in critically ill septic patients with SAE than in non-SAE patients 11 Higher blood levels in SAE patients than in healthy controls 13
IL-10	T-cells, B-cells, monocytes, macrophages	Protein	Blood	Lower plasma levels in CAP-associated sepsis with SAE than in non-SAE 14 Higher blood levels in SAE patients than in healthy controls 13
Colony-stimulating factor 3 (CSF3)	Endothelium, immune cells	Protein	Blood	Higher levels in critically ill septic patients with SAE than in non-SAE patients 10
Regulated on activation, normal T expressed and secreted (RANTES)	T cells, monocytes, epithelial cells	Protein	Blood	Lower plasma levels in CAP-associated sepsis with SAE than those with non-SAE 14
**Biomarkers of endothelial activation and/or damage**				
Intercellular adhesion molecule 1 (ICAM-1)	Endothelial cells	Glycoprotein	Blood	Higher soluble ICAM-1 levels in SAE than in non-SAE 30
Vascular cell adhesion molecule 1 (VCAM-1)	Endothelial cells	Glycoprotein	Blood	Higher soluble VCAM-1 levels in SAE than in non-SAE 30
Amino-terminal C-type natriuretic propeptide (NT-pro-CNP)	Endothelial cells	Propeptide	Blood, CSF	Plasma levels are increased in SAE, reaching a peak in early stage of SAE; CSF levels not different between SAE and non-SAE patients 28
Tumor necrosis factor receptor-associated factor 6 (TRAF6)	Endothelial cells	Protein	Blood	Higher blood levels patients with SAE compared to non-SAE 15
**Biomarkers derived from astrocytes or glial cells**				
S100β	Astrocytes	Protein	Blood	Higher blood levels patients with SAE compared to non-SAE 12 15 24 28 Serum levels of S100β did not correlate with the severity of neurological dysfunction in sepsis 25
Calcium-binding protein A8 (S100 A8)	Macrophages, dendritic cells, microvascular endothelial cells	Protein	Blood, CSF	Higher blood levels in patients with SAE compared to non-SAE 10 15 28 CSF levels not different in SAE and non-SAE 28
Glial fibrillary acidic protein (GFAP)	Astrocytes, nonmyelinating Schwann cells, enteric glial cells	Protein	Blood	Higher levels in SAE patients than in non SAE patients 22
Matrix metalloproteinases (MMPs)	Microglia	Protein	Blood	Higher levels of MMP8 in critically ill septic patients with SAE compared to non-SAE patients 10
Soluble triggering receptor expressed on myeloid cells 2 (sTREM2)	Microglia	Protein	CSF, blood	Higher CSF levels in SAE patients compared to healthy controls; no difference in blood levels 20
**Biomarkers reflecting neuronal injury**				
Neuron-specific enolase (NSE)	Neuronal body	Enzyme	Blood, CSF	Plasma NSE levels were not significantly different between SAE and nonseptic patients 28 CSF levels are increased in SAE compared to non-SAE patients 28 Plasma levels of NSE higher in SAE than in septic patients without SAE 15 24
Ubiquitin C-terminal hydrolase-L1 (UCH-L1)	Neuronal cytoplasm	Protein	Blood	Higher levels in SAE patients than in non-SAE patients 22
Tau protein	Neuronal cytoplasm, axons	Protein	Blood	Serum levels in septic patients with SAE significantly higher than in non-SAE patients 27
β-amyloid peptide (Aβ)	Axons	Peptide	Blood	Similar plasma levels in patients with SAE and healthy controls 13
Brain-derived neurotrophic factor (BDNF)	Axons	Protein	Blood	Higher plasma levels in CAP-associated sepsis with SAE compared to non-SAE 14
Neurofilament light chain (Nf-L)	Large-caliber myelinated axons	Protein	CSF, blood	Higher CSF levels in SAE patients compared to healthy controls 20 The levels and the increase over time of plasma Nf-L were significantly higher in sepsis patients with SAE compared to patients without SAE 21
**Biomarkers reflecting dysregulation of brain neurotransmitters**				
Erythrocytic acetylcholinesterase activity (AChE-activity)	Erythrocyte membrane	Enzyme	Blood	Significant changes over consecutive measurements in patients with SAE 32
**Biomarkers reflecting metabolism changes**				
4-hydroxyphenylacetic acid (4-HPA)	Colon	Phenol	Blood	4-HPA levels were higher in patients with SAE compared to controls 33
Ammonia	Colon, intestines	Hydrogen amine	Blood	Nonhepatic hyperammonemia correlated with an increased risk of SAE 34

Abbreviations: CAP, community acquired pneumonia; CSF, cerebrospinal fluid; IL, interleukin; SAE, sepsis-associated encephalopathy.

**Table 2:** Characteristics and results of included studies

Study	Study design	SAE ( *n* )	Non-SAE ( *n* )	Comparator	Population studied	Biomarker performed	Source of biomarker sampling	Measurements points	Disease severity	Mortality	Relevance of biomarker for SAE diagnosis	Relevance of biomarker for outcome
Nguyen et al 26	Prospective	27	143	Septic patients without SAE	27 SAE, 143 non-SAE	S100β, NSE	Blood	4 times	NR	NR	Increased S100β levels in 52% and increased NSE levels in 70% of SAE	Only S100β correlated with ICU mortality
Piazza et al 25	Prospective	21	0	No	21 SAE	S100β	Blood	3 times	Mean SOFA at ICU admission: 10.4	At 28 d: 42%	S100β increased in sepsis compared to NV, but not correlated with neurological dysfunction	S100β was not correlated with GCS, survival, or with neurological recovery
Yao et al 24	Cross-sectional	48	64	Septic patients without SAE	48 SAE, 64 non SAE	S100β, NSE	Blood	1 time	Median APACHEII in SAE: 22 and in non-SAE: 13	In hospital: 62.5% SAE, 23.4% non-SAE	S100β and NSE levels in SAE patients were significantly higher than those in non-SAE patients	Lower S100β levels in nonsurvivors
Su et al 30	Prospective	23	47	Septic patients without SAE	23 SAE, 47 non-SAE	sICAM-1, sVCAM-1, sE-selectin, sL-selectin, and sP-selectin	Blood	3 times	APACHE II in SAE: 21.3 ± 5.5 and in non-SAE: 17.5 ± 5.7 SOFA in SAE 8.2 ± 2.4 and non-SAE: 5.4 ± 3.1	In hospital: 40% SAE, 11% non-SAE	Serum sVCAM1, sICAM-1 levels in day 1 were higher in SAE than in non-SAE	NR
Zhang et al 15	Cross-sectional	29	38	Sepsis patients without SAE, healthy control	29 SAE, 28 non-SAE, 10 HC	S100A, TRAF6, S100β, NSE	Blood	1 time	APACHE II in SAE: 20.79 ± 9.07 and in non-SAE: 12.04 ± 3.93	At 28 d: 65.52% SAE, 17.86% non-SAE	S100A8, TRAF6 blood levels in SAE were significantly higher than those in non-SAE, and in non-SAE patients than in healthy controls	Higher S100A8, TRAF6 in patients who did not survive beyond 28 d
Tomasi et al 14	Cross-sectional	10	28	Septic patients without SAE, non-septic patients with delirium	10 SAE, 20 septic patients non-SAE, 8 nonseptic patients with delirium	BDNF, RANTES, IL-10	Blood	1 time	NR	NR	SAE had lower IL-10 and RANTES and higher BDNF levels compared to septic patients without SAE; plasma levels of RANTES and BDNF were significantly higher in patients with SAE compared to nonseptic patients with delirium	NA
Zhu et al 33	Cross-sectional	31	60	Healthy controls paired with SAE; septic patients without SAE	31 SAE, 44 controls, 16 sepsis non-SAE	4-HPA	Blood	1 time	NR	NR	4-HPA levels higher in patients with SAE compared to controls	4-HPA levels correlate with APACHE II, III, SOFA, and GCS
Wu et al 22	Prospective cohort	58	47	Septic patients without SAE	58 SAE, 47 sepsis without SAE	GFAP, UCH-L1	Blood	1 time	Maximum SOFA score: 10 ± 5	At 28 d: 31.77%	GFAP and UCH-L1 levels in SAE patients were significantly higher than those in non-SAE patients	GFAP and UCH-L1 levels correlated with GCS, APACHE II, SOFA scores, and higher mortality
Ehler et al 28	Prospective cohort	12	9	Nonseptic patients without encephalopathy	12 SAE, 9 nonseptic controls	NT-pro-CNP, NSE, S100β	Blood, CSF	3 times from blood, 1 time from CSF	NR	At 100 d: 41.6%	Plasma S100B and NT-pro-CNP higher in SAE compared to non-SAE, CSF levels were similar; NSE was higher in CSF of SAE patients than in non-SAE patients	No correlation of NT-pro-CNP, S100B, or NSE with outcome in patients with sepsis
Zhao et al 27	Retrospective	27	82	Septic patients without SAE	27 SAE, 82 non-SAE	Tau protein	Blood	1 time	APACHE II in SAE: 21.8 ± 6.7 and in non-SAE: 15.2 ± 4.6 SOFA in SAE: 8.7 ± 2.6 and in non-SAE: 4.8 ± 3.3	At 28 d, SAE: 51.9%, non-SAE: 26.8%	Serum tau protein level in SAE significantly higher than that in non-SAE	Tau protein level was significantly higher in nonsurvivors than in survivors at 28 d
Ehler et al 21	Prospective cohort	18	7	Patients without sepsis, patients with sepsis without SAE	18 SAE, 5 patients without sepsis, 2 patients with sepsis without SAE	Nf-L	Blood, CSF	CSF 1 time, blood 3 times	NR	NR	Plasma Nf-L values were significantly higher in patients with sepsis than in patients without sepsis and in and SAE compared to non-SAE	Plasma Nf-L values were significantly correlated with the severity of SAE (assessed by ICDSC values) and with a poorer functional outcome after 100 d; CSF Nf-L levels were increased in nonsurvivors compared with survivors
Orhun et al 13	Prospective	86	33	Healthy control	86 SAE, 33 healthy controls	IL-8, IL-6, IL-10, TNF-α, IL-12, C4b, C5a, and iC3b;	Blood	1 time	NR	NR	Increased IL-8, IL-6, IL-10, C4d, and decreased TNF-α, IL-12, C5a, and iC3b in SAE as compared to healthy controls	NR
Zujalovic et al 32	Cross-sectional	40	130	Nonseptic patients with and without delirium	40 SAE, 130 nonseptic patients (36 with delirium, 94 without delirium)	AChE activity	Blood	6 times	Median SAPSII 35.4 (sepsis group), 26.8 (nonsepsis group)	48.9% in the septic group, 7.7% in the nonseptic group	No change in AChE activity in nonseptic patients even with delirium; over 5 consecutive days statistically significant changes occurred compared to baseline in septic patients with SAE	No correlation of AChE activity with age, gender, SAPS II, SOFA score, delirium/SAE, or cognitive dysfunction in septic patients
Zhao et al 34	Retrospective	156	109	Septic patients without SAE	156 SAE, 109 non SAE	Ammonia	Blood	1 time	Median SOFA 6	NR	Hyperammonemia was associated with SAE	Patients with hyperammonemia had higher rates of short- and long-term mortality; ammonia levels correlated with GCS
Wu et al 12	Prospective cohort	59	45	Septic patients without SAE	59 SAE, 45 non-SAE	S100β	Blood	2 times	Median APACHEII in SAE: 20 and in non-SAE: 17; median SOFA in SAE: 11 and in non-SAE: 5	At 28 d, SAE: 45.7%, non-SAE: 11.1%	Serum S100β levels on days 1 and 3 in SAE compared to non-SAE	Increasing S100β levels were correlated with higher incidence of SAE, and higher 28- and 180-d mortality
Dong et al 10	Cross-sectional	10	21	Noninfected critically ill patient	10 SAE, 21 non SAE	IL-6, MMP8, CSF3, S100A8	Blood	1 time, 24 h after ICU admission	Mean APACHE II scores of 9.61 (non-SAE) and 14 (SAE) Mean SOFA of 4.90 (non-SAE) and 8.50 (SAE)	0 in both SAE and non-SAE	SAE patients had higher levels of IL-6, MMP8, CSF3, and S100A8 than non-SAE; statistically significant increase in SAE only for MMP8 and S100A8	S100A8 levels correlated with 28-d mortality, IL-6 levels correlated with the duration of mechanical ventilation
Mao et al 11	Cross-sectional	19	24	Noninfected critically ill patients	19 SAE, 24 non-SAE	IL-1B, IL-2, IL-4, IL-5, IL-6, IL-8, IL-10, IL-12p70, IL-17, IFN-α, IFN-γ, and TNF-α)	CSF	1 time	NR	15.79% in SAE	IL-6 and IL-8 levels were significantly elevated in SAE patients, with IL-8 having a better accuracy than IL-6	NA
Orhun et al 20	Cross-sectional	11	15	Age- and gender-matched control	11 SAE, 15 healthy controls	sTREM2, Nf-L	CSF, blood	1 time	Mean SOFA at ICU admission in SAE: 8.1 ± 3.9	18.18%	CSF levels of sTREM2 and Nf-L were significantly higher in SAE patients than in controls; serum sTREM2 levels were comparable in SAE and controls	sTREM2, Nf-L not correlated with the duration of hospitalization, APACHE II, SOFA, and SAPS II

Abbreviations: 4-HPA, 4-hydroxyphenylacetic acid; AChE-activity, erythrocytic acetylcholinesterase-activity; APACHE II, Acute Physiology and Chronic Health Evaluation II; BDNF, brain–derived neurotrophic factor; GCS, Glasgow Coma Scale; GFAP, glial fibrillary acidic protein; IFN-α, interferon-α; MMP8, matrix metalloproteinase 8; n, number; Nf-L, neurofilament light chain; NR, not reported; NSE, neuron-specific enolase; NT-pro-CNP, amino-terminal C-type natriuretic propeptide; NV, normal values; sICAM-1, soluble intercellular adhesion molecule 1; S100A8, calcium-binding protein A8; SAE, sepsis-associated encephalopathy; SOFA, Sequential Organ Failure Assessment; sTREM2, soluble triggering receptor expressed on myeloid cells 2; sVCAM-1, soluble vascular cell adhesion molecule 1; TNF-α, tumor necrosis factor-α; TRAF6, tumor necrosis factor receptor-associated factor 6; UCH-L1, ubiquitin C-terminal hydrolase-L1.

### Risk of Bias Assessment

As the study included only observational studies, risk of bias assessments were performed according to the Risk of Bias in Non-Randomized Studies of Interventions (ROBINS-I) tool, which consists of seven domains: confounding factors, selection of participants into the study, classification of interventions, deviations from intended interventions, missing data, measurement of the outcome, and selection of the reported result. 9 Each domain was graded as having low, moderate, serious, or critical risk of bias. In 12 of the 18 studies included, the overall risk of bias was considered moderate, in 5 studies it was assessed as serious, and 1 study was estimated to have a critical risk of bias. The quality of studies included according to the evaluation by ROBINS-I tool is detailed in Table 3 and in Supplementary File 2 (available in the online version only).

**Table 3:** Risk of bias assessment using the ROBINS-I tool for the included studies

Domain	Piazza et al 25	Wu et al 22	Ehler et al 21	Su et al 30	Nguyen et al 26	Tomasi et al 14	Orhun et al 19	Orhun et al 20	Mao et al 11	Wu et al 12	Dong et al 10	Ehler et al 28	Yao et al 24	Zujalovic et al 32	Zhu et al 33	Zhang et al 15	Zhao et al 34	Zhao et al 27
1. Bias due to confounding	S	M	M	M	M	M	M	S	M	M	M	M	M	M	M	M	M	M
2. Bias in selection of participants	M	L	L	L	L	L	L	M	M	L	M	L	L	L	M	L	M	L
3. Bias in classification of interventions	L	L	L	L	L	L	L	L	L	L	L	L	L	L	L	L	L	L
4. Bias due to deviations from intended interventions	L	L	L	L	L	L	L	L	L	L	L	L	L	L	L	L	L	L
5. Bias due to missing data	M	S	S	L	L	L	S	C	L	L	L	S	L	M	L	L	M	L
6. Bias in measurement of outcomes	S	M	M	M	M	M	M	S	L	M	M	M	M	M	M	M	L	M
7. Bias in selection of the reported result	L	L	L	L	L	L	L	L	L	L	L	L	L	L	L	L	L	L
8. Overall risk of bias	S	S	S	M	M	M	S	C	M	M	M	S	M	M	M	M	M	M

Abbreviations: C, critical; L, low; M, moderate; S, serious.

### Types of Biomarkers and Their Usefulness for the SAE Diagnosis and Prognosis Prediction

#### Cytokines

In a recent study, Dong et al reported significantly higher serum levels of matrix metalloproteinase-8 (MMP8) and calcium-binding protein A8 (S100A8) in critically ill septic patients with SAE compared to non-SAE patients, while the increase of other biomarkers studied (interleukin-6 [IL-6] and colony-stimulating factor 3 [CSF3]) in patients with SAE did not reach statistical significance. 10 All four biomarkers studied (S100A8, MMP8, CSF3, and IL-6) were significantly linked with the GCS scores of septic patients. 10 The S100A8 and MMP8 levels were helpful for the diagnosis of SAE, with S100A8 having a better prediction value than MMP8 (area under the receiver operating characteristic [AUC ROC] curve of 0.962 and 0.791, respectively). When a cutoff value of 82.29 pg/mL was considered, S100A8 had a sensitivity of 80% and specificity of 100% for the diagnosis of SAE. In this study, the S100A8 levels correlated significantly with the 28-day mortality of critically ill septic patients. 10

Mao et al assessed a panel of 12 inflammatory cytokines and chemokines (including IL-1B, IL-2, IL-4, IL-5, IL-6, IL-8, IL-10, IL-12p70, IL-17, IFN-α, IFN-γ, and tumor necrosis factor-α [TNF-α]) from the cerebrospinal fluid (CSF) of patients with SAE. They compared them with the levels found in nonseptic critically ill patients. 11 The results revealed that IL-8 was a better predictor of SAE than IL-6 with AUC ROC = 0.882 (95% confidence interval [CI] = 0.775–0.988) and 0.824 (95% CI = 0.686–0.961), respectively. The IL-8 levels did not correlate with the SAE patients' GCS scores. 11 Other studies reported higher IL-6 levels in patients with SAE compared with septic patients without SAE. 12

In their study comparing sepsis patients with SAE and matched healthy controls, Orhun et al reported significantly increased IL-8, IL-6, and IL-10 and significantly decreased TNF-α and IL-12 in patients with SAE as compared to healthy controls. 13 The levels of complement breakdown and activation products studied, such as C4d, were also increased, while C5a and iC3b showed decreased levels in patients with SAE compared to healthy individuals. 13

Blood biomarkers reflecting inflammation, endothelial activation, and neuronal injury were assessed and compared in patients with sepsis due to community-acquired pneumonia with and without SAE. 14 From all the biomarkers studied, the most useful to discriminate between patients with and without SAE were brain-derived neurotrophic factor (BDNF), regulated on activation normal T-cell expressed and secreted protein (RANTES), and IL-10. 14 Patients with SAE had lower plasma levels of IL-10 and RANTES and higher levels of BDNF compared to septic patients without SAE; the plasma levels of RANTES and BDNF were significantly higher in patients with SAE compared to patients without sepsis having delirium. 14

In summary, as neuroinflammation is one of the pathophysiologic mechanisms involved in SAE, several pro- and anti-inflammatory cytokines reflecting inflammation and oxidative stress may help detect sepsis-induced brain injury. Several articles focus on the correlation between SAE diagnosis, patients' outcomes, and inflammation-related biomarkers from the interleukin family measured in plasma or CSF. 10 11 13 14 Recent articles utilize bioinformatics to identify potential SAE biomarkers and validate their findings through clinical research. 10 11 Interestingly, specific biomarkers reflecting inflammation can differentiate between septic patients with or without neurological dysfunction and between different types of neurological dysfunction, for example, patients with SAE from nonseptic patients with delirium. 14 Therefore, biomarkers that indicate are a promising area for developing convenient diagnostic tools for SAE.

#### Neuronal and Glial Biomarkers of Brain Damage

In a study published in 2016, Zhang et al reported higher blood levels of proteins S100A8, S100β, neuron-specific enolase (NSE), and tumor necrosis factor receptor-associated factor 6 (TRAF6) in patients with SAE compared to non-SAE patients. 15 The levels of S100A8 and TRAF6 were also higher in septic patients without SAE compared to healthy controls. 15 S100β is expressed in astrocytes, while S100A8 is primarily expressed by microglial cells, which are among the first cells involved in immune defense. This likely explains why S100A8 has better specificity for SAE diagnosis compared to S100β. 15 S100A8 levels of 1.93 ng/mL predicted SAE with 92.90% specificity and 69.00% sensitivity, with an AUC ROC of 0.860, while S100β levels of 0.28 µg/L had 68.80% specificity and 83.30% sensitivity for SAE diagnosis, with an AUC ROC of 0.790. 15 TRAF6 is a member of the TRAFs superfamily of membrane adapter proteins, essential for intracellular signaling in immune and nonimmune cells. 16 NSE is a glycolytic isoenzyme mostly found in mature neurons and oligodendrocytes, and its release is associated with acute brain injury. 17 Zhang et al found that S100A8 demonstrated better specificity for SAE diagnosis and mortality prediction compared to S100β and TRAF6 despite having lower sensitivity. 15 NSE had similar specificity as TRAF6 for SAE diagnosis prediction but lower sensitivity. 15

Neurofilament light chain (Nf-L), a component of a complex called neurofilament, is a protein found principally in large-caliber myelinated axons of central and peripheral nervous system. 18 With axonal damage, it is released into the CSF and blood. 19 A recent study reported significantly higher CSF levels of soluble triggering receptor expressed on myeloid cells 2 (sTREM2) and Nf-L and similar serum sTREM2 levels in patients with SAE than in age-matched controls. 20 The microglia is the primary source of sTREM2. In the same study, the significant correlation between CSF sTREM2 and Nf-L levels suggests the relation between glial hyperactivity and neuroaxonal damage in SAE. 20 Although these biomarkers could be used for diagnosing SAE, their usefulness for follow-up and outcome prediction in patients with sepsis and septic shock is limited. The levels of sTREM2 in blood were not different between SAE and controls and did not correlate with CSF levels despite the BBB disruption described in SAE.

Ehler et al evaluated the levels of Nf-L from the CSF and blood in a pilot study including 20 patients with sepsis and 5 patients without sepsis, and found that plasma Nf-L levels were higher in sepsis compared to patients without sepsis, having a significant increase from day 1 to day 7 only in septic patients. 21 The increase in plasma Nf-L levels over time was significantly greater in sepsis patients experiencing delirium, as assessed by the CAM-ICU score, compared to those without delirium. This increase also correlated with the extent of MRI findings suggesting SAE. 21 In terms of prognosis prediction, plasma Nf-L values were strongly associated with the severity of SAE and correlated with poorer functional outcomes after 100 days. Additionally, the CSF Nf-L levels were higher in nonsurvivors compared to survivors. 21

Another study suggested that serum-based neuronal and glial proteins (glial fibrillary acidic protein [GFAP], and ubiquitin carboxyl-terminal hydrolase-L1 [UCH-L1]) could be valuable for diagnosing SAE, predicting outcomes, and assessing long-term quality of life in patients with sepsis. 22 Both GFAP and UCH-L1 levels could discriminate between septic patients with and without SAE, with optimal cutoff values of 0.532 and 7.72 ng/mL, respectively, and AUC ROC of 0.824 (95% CI: 0.738–0.892) and 0.812 (95% CI: 0.724–0.881), respectively. 22 In response to hypoxia, stress, and inflammation, septic patients experience increased apoptosis and structural changes in specific brain areas, such as the amygdala, nuclei tractus solitarii, and locus ceruleus. This process explains the release of neuronal and glial proteins into the bloodstream due to the compromised BBB, which is associated with neurological dysfunction. 22 23

Another study reported significantly higher S100β and NSE levels in SAE patients compared to patients without SAE. However, only S100β was correlated with the severity of brain dysfunction and could predict the outcome of septic patients. 24 While S100β demonstrated a good sensitivity but low specificity for SAE diagnosis, NSE had better specificity, but the efficacy and sensitivity were weak. 24 Interestingly, none of the two biomarkers could differentiate between the two main types of SAE (type A manifested with agitation, confusion, irritability, seizures, or type B with somnolence, stupor, and coma). 24 These results contradict a previous study by Piazza et al that reported increased serum levels of S100β compared to reference ranges, without correlation with the severity of neurological dysfunction or the patient's neurological outcome in a small group of sepsis patients with SAE. 25 Nguyen et al reported increased S100β levels in 52% and increased NSE levels in 70% of their patients with SAE, with a correlation of S100β levels with type B encephalopathy. In contrast, no correlation with encephalopathy type was reported for NSE levels. 26 In the same study, only increased S100β levels were associated with ICU mortality, while GCS scores or NSE levels were not helpful for outcome prediction. 26 Wu et al measured the levels of S100β in septic patients on days 1 and 3 after ICU admission and found higher S100β in both measurements in septic patients with SAE than in non-SAE. However, only the S100β levels on day 3 independently correlated with SAE after adjusting for confounders such as disease severity and sex. 12 This study reported a stronger correlation between GCS scores and S100β levels on day 3 compared to day 1. It also found a higher incidence of encephalopathy, as well as increased mortality rates at 28 and 180 days in septic patients whose S100β levels rose from day 1 to day 3. These dynamic changes in S100β levels are useful for diagnosing SAE and predicting patient outcomes. 12 A study found higher S100β levels and similar NSE and amyloid β peptide plasma levels in patients with SAE compared to healthy controls. 13

According to the study by Zhao et al, serum tau protein levels serve as a promising biomarker. Specifically, when the tau protein level exceeds 71.96 pg/mL, it can predict the development of SAE with 70.4% sensitivity and 72.0% specificity and an AUC ROC of 0.770, with a 95% CI of 0.671 to 0.869. 27 In the septic patients studied, the ability to predict SAE using tau protein levels was found to be more effective than using the Sequential Organ Failure Assessment (SOFA) score. 27 Additionally, tau protein levels were significantly higher in nonsurviving patients at 28 days compared to those who survived, indicating its potential value in predicting outcomes. 27

These findings show the usefulness of biomarkers reflecting microglial, astroglial activation, and neuronal injury. The release of neuron-expressed proteins indicates ongoing neurological damage, as they can pass through the damaged BBB and be measured in blood. Astroglial biomarkers, such as proteins from the S100 family, are among the most well-studied biomarkers for SAE diagnosis. They have a high accuracy for SAE prediction and may also indicate the outcome of the septic patients. 10 12 15 24 25 26 28 However, the reported sensitivity and specificity for SAE diagnosis are variable in different studies, depending on the population included and the biomarker cutoff levels considered. The absolute values and changes of these biomarkers over time are valuable; for example, S100β has a short half-life, and the levels decrease rapidly when the brain damage ceases. Therefore, it is a valuable biomarker that reflects the dynamic changes seen in SAE. 12

#### Biomarkers of Endothelial Activation and Damage

The expression of adhesion molecules such as intercellular adhesion molecule 1 (ICAM-1), vascular cell adhesion molecule 1 (VCAM-1) increases on the endothelial cells at the level of BBB in sepsis. 8 29 In their study, Su et al measured the serum levels of soluble ICAM-1 (sICAM-1) and soluble VCAM-1 (sVCAM-1) in patients with sepsis after 1, 3, and 7 days since sepsis diagnosis and found that only serum sVCAM-1 measured on day 1 correlated independently with SAE after adjusting for confounders. 30 The suggested cutoff value of sVCAM-1 on day 1 for predicting SAE was 1,900 ng/mL, resulting in a sensitivity of 81.8% and a specificity of 61.9%, with an AUC ROC of 0.760 for SAE diagnosis. 30 Interestingly, in this study, the sVCAM-1 level gradually decreased from day 1 to 7 both in patients with and without SAE but remained higher in SAE in all three different testing points. 30 Therefore, according to Su et al, the sVCAM-1 level on presentation is a better SAE predictor than other adhesion molecules. 30

Another biomarker relevant for diagnosing SAE is the amino-terminal C-type natriuretic propeptide (NT-pro-CNP) that is produced in different tissues, but having the highest concentrations in the brain. 28 In their study, Ehler et al reported higher plasma levels of S100B and NT-pro-CNP in SAE patients compared to non-SAE, with similar CSF levels between the groups. In SAE patients, the NSE levels increased in the CSF, while the plasma values were similar across the groups. 28 Interestingly, mean CSF NT-pro-CNP levels were higher in patients with septic brain lesions on MRI compared to those without brain lesions. 28 The relationship between inflammation and the release of NT-pro-CNP in CSF is supported by the correlation between elevated levels of CSF NT-pro-CNP and IL-6, and plasma procalcitonin levels. 28 NT-pro-CNP is considered a promising biomarker for SAE because its levels peak in the early stages of sepsis. This means it can help predict the early development of SAE and has a long life in circulation. 8 28

These findings reveal the relevance of biomarkers of endothelial activation for SAE diagnosis. Systemic inflammation leads to endothelial activation and damage involving the BBB, primarily composed of microvascular endothelial cells. Endothelial dysfunction is known to occur early, so endothelial-related biomarkers are among the earliest to increase in sepsis. 8

##### Other Markers

Changes in brain neurotransmitters, such as acetylcholine, resulting in disruption of central cholinergic activity have been described as a possible pathophysiological mechanism contributing to SAE. 31 However, the direct measurement of acetylcholine is not feasible due to the rapid degradation in the synaptic cleft, and the erythrocytic acetylcholinesterase activity (AChE-activity) can be used as a surrogate parameter of central cholinergic transmission. The decrease of neurotransmitter acetylcholine due to the damage of cholinergic neurons leads to altered activity of the surrogate parameter AChE-activity. In their study, Zujalovic et al demonstrated a statistically significant change in AChE-activity for at least 5 consecutive days compared to baseline in septic patients with SAE, while no significant changes in AChE-activity were reported in nonseptic patients, even in those with delirium. 32 According to their results, the longitudinal measurement of AChE activity could be helpful in the diagnosis of SAE.

Another study reports changes in plasma metabolites in patients with SAE, the most relevant finding being increased plasma levels of 4-hydroxyphenylacetic acid in SAE compared to matched controls. 33 The levels of this metabolite also correlated with severity scores (Acute Physiology and Chronic Health Evaluation [APACHE], SOFA) and GCS. 33 Nonhepatic hyperammonemia was correlated with GCS score and an increased risk of SAE in sepsis. 34 According to the study by Zhao et al, the blood ammonia level was an independent risk factor for long-term prognosis in patients with sepsis. 34

## Discussion

The diagnosis of SAE is challenging since it is an exclusion diagnosis mainly based on clinical neurological examination of the patients. The challenges are linked to (1) the wide range of clinical symptoms of SAE, (2) the requirement to rule out other neurological disorders or causes of metabolic encephalopathy, and (3) the lack of specificity in additional diagnostic tests that may be employed.

Not only the clinical manifestations of SAE may be highly variable, from agitation or mild delirium to seizures and coma, but also the time of SAE occurrence can differ depending on the severity of the infection and patient characteristics. Additionally, the clinical evaluation of patients with sepsis can be challenging, especially in critically ill patients who present with associated conditions such as fever or hypothermia, pain, acid–base, and electrolyte disturbances or are sedated to facilitate mechanical ventilation or different procedures. Therefore, it is easy to understand why the neurological examination of those patients and excluding contributing factors other than sepsis is challenging. In clinical settings, neurologists frequently encounter the difficulty of determining whether the sudden appearance of neurological symptoms is caused by a primary CNS condition (like stroke or meningitis/encephalitis) or it is secondary to sepsis. This distinction is critical, as any delay in diagnosing the issue can result in severe consequences. Unfortunately, to date, there is no simple measurement or validated biomarker that can distinguish between a primary CNS condition and SAE.

Other investigations may be added to clinical examinations or in the cases where the clinical neurologic examination cannot be performed or is incomplete. Imaging techniques such as CT or MRI, transcranial Doppler ultrasonography, and EEG can be used to diagnose SAE. However, the results of these investigations lack specificity for SAE, and there are no clear recommendations on which investigation to perform to establish the diagnosis of SAE. Moreover, some of these investigations require patient transportation outside the ICU (CT and MRI), which is not feasible and can be dangerous in case of unstable patients; they are time-consuming, their results can be influenced by sedation (EEG), and the equipment needed is costly and not available in many places.

SAE is a common finding in sepsis patients, with a reported incidence of more than 70% depending on the studied population. 1 2 4 The diagnosis of SAE is essential in clinical practice, as it is associated with higher short-term mortality and long-term complications. 1 35 Sometimes, the neurological dysfunction manifests early and represents the first organ dysfunction. Thus, early identification of SAE may be crucial for prompt sepsis diagnosis and high-risk patient recognition. Therefore, diagnosing SAE using biomarkers would be valuable in clinical practice, allowing testing independent from sedative medications and decreasing the need for sedation breaks or patient transport outside the ICU for imagistic investigations. In medicine, biomarkers are largely used as measurable indicators for certain diseases, for example, troponin for acute coronary syndromes, serum creatinine for renal failure, or cancer biomarkers. Depending on the biomarkers used and techniques developed, this approach may reduce the cost of the differential diagnosis of encephalopathy in septic patients and patient care. Moreover, selected biomarkers may be more sensitive for SAE detection and less time-consuming than neuropsychiatric examination and repetitive assessment using delirium scales or brain MRI. 10

According to the findings from the articles reviewed, promising biomarkers exist for diagnosing and predicting the prognosis of SAE. These biomarkers reflect various factors, including inflammation, endothelial activation and damage, astrocytic and microglial activation, neuronal injury, and metabolism changes. When compared between studies, the reported sensitivity and specificity of the biomarkers for diagnosing SAE varied depending on the population included and the biomarker cutoff levels considered. When assessing sepsis-induced brain injury, evaluating extracellular secretory proteins or biomarkers reflecting neuronal damage in CSF may have better sensitivity and specificity for an early diagnosis of SAE than blood biomarkers. However, assessing CSF biomarkers is more difficult in clinical practice, and since the BBB is disrupted early in the septic process, protein structures can pass through, and their blood levels parallel the CNS levels.

As SAE has a complex pathophysiology, depends on various factors related to both the infectious disease and the patient, and has different degrees of severity and dynamic evolution, future research should consider that a panel of biomarkers may be more appropriate for diagnosis than a single one. Also, since brain injury in sepsis is an evolving process, the trend over time of biomarkers may be valuable not only for diagnosis but also for predicting prognosis.

Since the SAE diagnosis is established mainly by clinical evaluation, most studies focused on the correlation between the clinical manifestations or the scoring systems for consciousness or delirium assessment and the biomarker levels for assessing the usefulness of biomarkers in SAE diagnosis. Such an approach is helpful. However, future studies should also assess the relationship between biomarkers and various investigations used for SAE diagnosis (CT, EEG, MRI). Future studies should also include patients with neurological disorders not related to sepsis and assess which biomarkers can differentiate between the acute neurological disturbances induced by sepsis and those resulting from other pathological processes.

The use of biomarkers for SAE diagnosis is still in the early stage of development. Currently, no specific and standardized biomarker is recommended for SAE diagnosis. Future studies need to address unanswered questions, such as the recommended panel of biomarkers, the timing of biomarker measurements, their cutoff values, specificity and sensitivity for SAE diagnosis, and their relation with SAE severity and patient outcomes.

## Conclusion

The early diagnosis of SAE may be improved by adding to the assessment methods already in use the screening of a panel of validated biomarkers. Various biomarkers may help diagnose and predict outcomes in SAE, but their usefulness in clinical practice remains limited. More research is needed to identify the biomarkers that can improve the SAE diagnosis, the moment when the biomarker levels should be assessed, and the accurate interpretation of the biomarker levels obtained in relation to SAE severity and patient outcomes.

## Notes

**Corrigendum:** A corrigendum has been published for this article (DOI: 10.1055/s-0046-1819691).
