# Corrigendum: The Diagnosis of Sepsis-Associated Encephalopathy Using Biomarkers: Are We There Yet?

**DOI:** 10.1055/s-0046-1819691

**Published:** 2026-03-27

**Authors:** Florin Scarlatescu, Ecaterina Scarlatescu, Dana R. Tomescu, Daniela Bartos

**Affiliations:** 1 Department of Neurology, Clinical Emergency Hospital, Bucharest, Romania; 2 University of Medicine and Pharmacy Carol Davila, Bucharest, Romania; 3 Department of Anaesthesia and Intensive Care, Fundeni Clinical Institute, Bucharest, Romania

It has been brought to the publisher's attention that author “Florin Scarlatescu” is not just affiliated with affiliation “Department of Neurology, Clinical Emergency Hospital, Bucharest, Romania” as shown in the currently published version of the article published in the Avicenna of Journal of Medicine, Volume 15, issue 2 (DOI: 10.1055/s-0045-1808060 ) but he was also affiliated, at the time of the study, with the University of Medicine and Pharmacy ‘Carol Davila’, Bucharest, Romania, as a PhD student. This has been corrected and reflects in the online published version.

