# Supplementary material for: The Diagnosis of Sepsis-Associated Encephalopathy Using Biomarkers: Are We There Yet?
Source: Avicenna J Med. 2025 May 6;15(2):51–63. doi: 10.1055/s-0045-1808060 (PMC12178668; doi:10.1055/s-0045-1808060)
Supplement: Supplementary file 1 — Supplementary Material [file 10-1055-s-0045-1808060-s240115.pdf]

Supplementary File 1 Search strategy of MEDLINE electronic database

((“sepsis associated encephalopathy”[Title/Abstract] OR “sepsis associated encephalopathy”[Title/Abstract] OR “septic encephalopathy”[Title/Abstract] OR ((“septic”[All Fields] OR “septics”[All Fields]) AND “neurologic dysfunction”[Title/Abstract]) OR “sepsis induced encephalopathy”[Title/Abstract] OR “sepsis induced encephalopathy”[Title/Abstract]) AND (“biomarker”[Title/Abstract] OR “laboratory test”[Title/Abstract] OR “plasmatic marker”[Title/Abstract] OR “blood test”[Title/Abstract] OR “serological marker”[Title/Abstract] OR “diagnosis”[Title/Abstract]) AND (“humans”[MeSH Terms] AND “english”[Language]) AND (“humans”[MeSH Terms] AND 1970/01/01:2024/12/31[Date - Publication] AND “english”[Language])) AND ((humans[Filter]) AND (1970:2024[pdat]) AND (english[Filter]))

Risk of Bias Assessment Using the ROBINS-I Tool for Studies on Biomarkers in Sepsis-Associated Encephalopathy

Assessment Rationale for Each Study

Piazza et al (2007): Main outcome—correlation between S100B levels and severity of encephalopathy

- **Confounding:** Serious risk. Inadequate adjustment for potential non-CNS sources of S100B protein despite acknowledging these sources. No multivariable analysis to control for confounding variables.
- **Selection:** Moderate risk. Only 21 of 75 sepsis patients during the study period were included with incomplete reporting of the selection process.
- **Outcome measurement:** Serious risk. Significant limitations in neurological assessment as GCS was used despite acknowledged problems with assessment in sedated patients. Only 5 of 21 patients had CT scans performed.
- The study was judged to have an **overall serious risk of bias**, primarily driven by limitations in handling confounding factors, missing data, and outcome measurement challenges.

Wu et al (2019): Main outcome—diagnostic value of GFAP and UCH-L1 for SAE

- **Confounding:** Moderate risk. Multivariate logistic regression was implemented, but the authors acknowledged that not all potential confounders were adequately addressed.
- **Missing data:** Serious risk. Substantial missing outcome data, with only 59 of 105 patients (56%) successfully followed up at 180 days.
- **Outcome measurement:** Moderate risk. Potential impact of sedation on SAE assessment was acknowledged, but efforts were made to mitigate through spontaneous awakening trials.
- The study was judged to have an **overall serious risk of bias**, primarily driven by limitations in handling confounding factors, missing data, and outcome measurement challenges.

Ehler et al (2019): Main outcome—prognostic value of neurofilament levels in sepsis patients

- **Confounding:** Moderate risk. Small sample size limiting the ability to adjust for all potential confounders, although strict exclusion criteria were applied.
- **Missing data:** Serious risk. Significant missing data for CSF samples, with only 12 of 20 sepsis patients (60%) having samples available for analysis.
- **Outcome measurement:** Moderate risk. Standard MRI examinations may have underestimated the extent of brain injury, as acknowledged by the authors.
- The study was judged to have an overall serious risk of bias, primarily driven by limitations in handling confounding factors, missing data, and outcome measurement challenges.

Su et al (2014): Main outcome—association between sVCAM-1 levels and septic encephalopathy

- **Confounding:** Moderate risk. The study adjusted for some potential confounders using stepwise logistic regression (final model included sVCAM-1, age, and maximum 24-hour SOFA score), but may not have controlled for all important confounding variables.
- **Selection of participants:** Low risk. Clear inclusion and exclusion criteria were defined. The study excluded patients with trauma, surgical treatment, hematologic diseases, pregnancy, preexisting CNS disorders, and those exposed to substances known to affect consciousness.
- **Missing data:** Low risk. The study does not explicitly mention missing data issues, and it appears that all enrolled patients had complete data for the primary analyses.
- **Outcome measurement:** Moderate risk. Septic encephalopathy was clearly defined, but assessment of neurological status might have some subjectivity. Short-duration sedatives were used when necessary, which could potentially affect assessments.

Nguyen et al (2006): Main outcome—association between biomarker levels and brain injury in sepsis

- **Confounding:** Moderate risk. Multivariate Cox regression was used to analyze various factors (age, GCS score, biomarker values, severity scores) on ICU survival, but may not have adjusted for all potential confounders.
- **Selection of participants:** Low risk. Well-defined inclusion and exclusion criteria. They excluded patients with primary CNS disorders, acute mental deterioration due to nonseptic causes, and sepsis within 2 weeks after events that could affect biomarker levels.
- **Missing data:** Low risk. All 170 patients appeared to have complete biomarker measurements with no significant missing data reported.
- **Outcome measurement:** Moderate risk. Encephalopathy was well defined as type A or B, but neurological assessment might be affected by sedation despite their statement that continuous sedation was never used. Brain imaging was only performed in a subset of patients.

Both Su et al and Nguyen et al were assessed to have an **overall moderate risk of bias**, primarily due to their more complete data collection and relatively stronger methodological approaches compared to the other studies with serious risk of bias. The domain-specific assessments reveal better handling of missing data and clearer selection criteria in these studies, though moderate concerns remain regarding confounding control and outcome measurement.

Tomasi et al (2016): Main outcome—differences in biomarker levels between SAE, sepsis without encephalopathy, and delirium groups

- **Confounding:** Moderate risk. Examined a homogeneous group of CAP-induced sepsis patients, which limits some confounding, but small sample size (30 sepsis patients, 10 with SAE, 8 nonseptic delirium patients) limited the ability to adjust for all potential confounders.
- **Missing data:** Low risk. No explicitly mentioned missing data issues, and all enrolled patients appear to have complete biomarker measurements.
- **Outcome measurement:** Moderate risk. Used standardized techniques for biomarker measurements, but potential bias in delirium assessment in patients requiring sedation.

Orhun et al (2019): Main outcome—association between inflammatory markers and cognitive outcomes in SAE

- **Confounding:** Moderate risk. Included 86 SAE patients but only 21 underwent cognitive assessment, limiting full adjustment for confounders.
- **Missing data:** Serious risk. Substantial missing data for cognitive outcomes, with only 21 of 86 patients (24%) undergoing cognitive assessment, likely favoring less severe cases.
- **Outcome measurement:** Moderate risk. Used standardized cognitive tests and biomarker measurement methods, but some assessments potentially affected by sedation.

Orhun et al (2023): Main outcome—levels of sTREM2 and Nf-L in CSF and association with cognitive outcomes

- **Confounding:** Serious risk. Very small sample size (11 SAE patients, 15 controls) severely limiting adjustment for confounders.
- **Missing data:** Critical risk. Extremely high missing data for cognitive outcomes with only 4 of 11 SAE patients (36%) having cognitive assessment data.
- **Outcome measurement:** Serious risk. While standardized measurement techniques were used, the very small sample size for cognitive outcomes ($n = 4$) severely limits reliability.

Mao et al (2023): Main outcome—identification of IL-8 as potential biomarker for SAE

- **Confounding**: Moderate risk - Combined bioinformatics analysis with clinical validation in 19 SAE patients and 24 controls, with limited adjustment for confounders.
- **Selection of participants:** Moderate risk. Selection criteria defined but the recruitment process from eligible population not fully described.
- **Missing data:** Low risk. No explicitly mentioned missing data issues; all enrolled patients appear to have complete cytokine measurements.
- **Outcome measurement:** Low risk. Used standardized techniques (flow cytometry) for measuring cytokine levels in CSF.

Two studies (Tomasi et al and Mao et al) were assessed to have an overall moderate risk of bias, with better data completeness and measurement approaches. Orhun et al was assessed to have critical risk of bias primarily due to the extremely small sample size ($n = 4$) for cognitive outcome assessment, which severely limits the reliability of findings related to cognitive outcomes.

Wu et al (2020): Main outcome—association between serum S100B levels and SAE diagnosis

- **Confounding:** Moderate risk. The study adjusted for some confounders using multiple logistic regression analysis, including APACHE II scores minus GCS scores. However, potential confounders such as the effect of sedation on S100B levels or other organ dysfunctions affecting S100B were not fully addressed.
- **Selection of participants:** Low risk. Clear inclusion and exclusion criteria were described. They excluded patients with primary brain injury and other factors that could affect S100B levels. Out of 173 screened patients, 104 were included with transparent reasoning.
- **Missing data:** Low risk. For the primary analysis of S100B association with SAE, all 104 patients had complete data, though there were some missing data for the quality of life assessment (only 60 of 104 patients had successful follow-up).
- **Outcome measurement:** Moderate risk. SAE was defined as GCS < 15 or at least one positive CAM-ICU score, which are standardized tools. However, the authors acknowledged limitations in using GCS in sedated patients.

Dong et al (2023): Main outcome—identification of extracellular proteins as biomarkers for SAE

- **Confounding:** Moderate risk. The study combines bioinformatics analysis with experimental validation in 31 sepsis patients (10 SAE, 21 non-SAE). For clinical validation, there is limited adjustment for potential confounders despite some baseline differences between groups.
- **Selection of participants:** Moderate risk. The inclusion criteria for the clinical validation part are described, but the selection process from the eligible population is not fully detailed. The bioinformatics part used existing datasets with limited information on the original patient selection.
- **Missing data:** Low risk. No explicitly mentioned missing data issues in the clinical validation part. All enrolled patients appear to have complete biomarker measurements.

 © 2025. The Author(s).

- **Outcome measurement:** Moderate risk. Used standardized techniques (ELISA) for measuring biomarker levels. However, while the diagnostic criteria for SAE were mentioned, the assessment methodology and potential observer bias were not described in detail.

Both studies (Dong et al and Wu et al) were assessed to have an overall moderate risk of bias, with their main strengths being in the domains of intervention classification, protocol adherence, and transparent reporting of results. The main limitations were in confounding control and potential bias in outcome measurement related to the challenges in neurological assessment of critically ill patients.

Ehler et al (2019): Main outcome—diagnostic value of NT-pro-CNP compared to NSE and S100B for sepsis-associated encephalopathy

- **Confounding:** Moderate risk. There is a significant age difference between the sepsis patients (mean age: 67.8 ± 12.1 years) and neurologic controls (mean age: 34.8 ± 13.1 years). The study found that CSF NT-pro-CNP levels correlated with patient age ($r = 0.760$, $p < 0.01$), which could confound results. The control group also consisted of subjects with neurological complaints rather than healthy controls.
- **Selection of participants:** Low risk. Clear inclusion and exclusion criteria were defined for both septic patients with SAE and neurologic controls. The exclusion criteria appropriately eliminated patients with preexisting CNS disease or conditions that might affect biomarker measurements.
- **Missing data:** Serious risk. MRI data were missing for 3 of 12 sepsis patients (25%). While biomarker measurements were reported as complete for all included participants, the small sample size (12 sepsis patients, 9 controls) limits the reliability of findings.
- **Outcome measurement:** Moderate risk. The assessment of SAE involved standardized methods including neuropsychiatric examination and CAM-ICU. However, the timing of CSF sampling varied (mean time from sepsis onset to lumbar puncture was 3.6 ± 1.8 days), which could affect biomarker levels. MRI assessment was conducted by a blinded neuroradiologist, which helps reduce measurement bias.

This study was assessed to have an overall serious risk of bias, primarily due to concerns about missing data and the small sample size, which limit the ability to adjust for confounding factors and affect the reliability of the findings.

Yao et al (2014): Main outcome—diagnostic and prognostic value of S100β and NSE for SAE

- **Confounding:** Moderate risk. The study controlled for some confounders by excluding patients with primary brain injury, but did not fully account for nonbrain sources of S100β that could influence levels during sepsis.
- **Selection of participants:** Low risk. Clear inclusion and exclusion criteria with consecutive enrollment of septic patients. They excluded patients with primary brain injury, cerebral stroke, epilepsy, intracranial infection, and other conditions.
- **Missing data:** Low risk. Complete data for S100β and NSE levels appear to be available for all included patients (112 septic patients, 48 with SAE).
- **Outcome measurement:** Moderate risk. While biomarker measurement used standardized laboratory techniques, SAE diagnosis relied partly on subjective assessments with acknowledged limitations in sedated patients.

This study by Yao et al examined S100β and NSE as biomarkers for sepsis-associated encephalopathy (SAE). The overall risk of bias is assessed as moderate, with the main strengths being clear participant selection criteria and standardized biomarker measurements. The primary limitations are potential unmeasured confounding and some challenges in assessing SAE in sedated patients. The findings on the diagnostic and prognostic value of S100β for SAE appear reasonably reliable, though confirmation in larger studies adjusting for additional confounders would strengthen the conclusions.

Zujalovic et al (2020): Main outcome—changes in AChE-activity over time in septic patients with SAE

- **Confounding:** Moderate risk. The study acknowledged potential influence of anticholinergic medications but could not exclude these factors. Sample size limited the ability to adjust for all confounders.
- **Selection of participants:** Low risk. Clear inclusion criteria with systematic enrollment process from 241 potentially eligible patients.
- **Missing data:** Moderate risk. Of 241 eligible patients, 66 were excluded with 38 having fewer than two AChE measurements due to short ICU stays, potentially biasing results if these patients differed systematically.
- **Outcome measurement:** Moderate risk. The authors acknowledged CAM-ICU limitations for SAE diagnosis and potential effects of sedation on neurological assessment despite mitigation efforts.

Zhu et al (2019): Main outcome—plasma metabolite biomarkers for SAE, focusing on 4-HPA

- **Confounding:** Moderate risk. The study matched by age, gender, and BMI, but small sample sizes in GCS subgroups (as few as 5 patients in some groups) limited the ability to control for confounders.
- **Selection of participants:** Moderate risk. Selection process from 47 septic patients to 31 SAE patients not fully detailed. Requirement for auxiliary tests (EEG, MRI, CT, or PCD) might have introduced selection bias.
- **Missing data:** Low risk. Metabolic profiling appears complete for all included patients without significant missing data reported.
- **Outcome measurement:** Moderate risk. Standardized GC-MS techniques were used, but GCS-based classification of SAE severity has limitations, especially for sedated patients.

 © 2025. The Author(s).

Both studies (Zujalovic et al and Zhu et al) were assessed to have an overall moderate risk of bias, with their main limitations being in handling potential confounding factors and some challenges in outcome measurement related to assessing neurological function in critically ill patients. However, they maintained reasonable methodological approaches to selection, classification, and outcome reporting.

Zhang et al (2016): Main outcome—diagnostic and predictive value of S100A8 and TRAF6 for SAE

- **Confounding:** Moderate risk. While they excluded patients with primary brain injury and other factors that could affect results, they acknowledge that "the severity of sepsis represents a risk factor for ICU delirium and a confounding factor for the relationship between SAE and S100A8 or TRAF6 levels.
- **Selection of participants:** Low risk. Clear inclusion and exclusion criteria with systematic selection process. They excluded patients with primary brain injury, intracranial infection, cerebral infarction, and other conditions that could affect results.
- **Missing data:** Low risk. The study does not explicitly mention missing data issues. All enrolled patients (57) appear to have complete biomarker measurements.
- **Outcome measurement:** Moderate risk. Standardized laboratory techniques were used for biomarker measurements, but they acknowledged limitations in measuring neurological dysfunction using GCS in sedated patients, noting they performed "spontaneous awakening trials daily to assess the consciousness."

Zhao et al (2020): Main outcome—association between nonhepatic hyperammonemia and mortality in sepsis

- **Confounding:** Moderate risk. The study adjusted for some potential confounders using multivariate analysis, but acknowledged limitations: "…information was missing on some clinical variables, such as bilirubin, albumin, and intravenous nutrition. Inclusion of those data may have led to a more comprehensive understanding."
- **Selection of participants:** Moderate risk. Clear inclusion and exclusion criteria, but retrospective selection from the MIMIC-III database using ICD-9 codes could introduce selection bias. Of 2,159 patients tested for blood ammonia, only 265 were included after screening.
- **Missing data:** Moderate risk. The authors acknowledge that "Variables with missing data are relatively common in the MIMIC-III database and we replaced them with median values." This imputation approach could introduce bias if missing data were not random.
- **Outcome measurement:** Low risk. Outcomes of mortality are objective measures reliably recorded in databases. The association with SAE was assessed using standardized clinical definitions including GCS scores and diagnosis of delirium/encephalopathy.

Both studies (Zhao et al and Zhang et al) were assessed to have an overall moderate risk of bias, with their main limitations being in handling potential confounding factors while maintaining reasonable methodological approaches to selection, classification, and outcome reporting.

 © 2025. The Author(s).
